# Supplementary material for: Development of Two Patient Self-Reported Measures on Functional Health Status (FOD) and Health-Related Quality of Life (QOD) in Adults with Oropharyngeal Dysphagia Using the Delphi Technique
Source: J Clin Med. 2022 Oct 7;11(19):5920. doi: 10.3390/jcm11195920 (PMC9572600; doi:10.3390/jcm11195920)
Supplement: Supplementary file 1 [file jcm-11-05920-s001.zip › jcm-1916787-supplementary.pdf]

Supplementary File S1.

Development of two patient self-reported measures on functional health status (FOD) and health-related quality of life (QOD) in adults with oropharyngeal dysphagia using the Delphi technique

Renée Speyer [1,2,3], Reinie Cordier [2,4], Deborah Denman [5], Catriona Windsor [1], Gintas P. Krisciunas [6], David Smithard [7] and Bas J. Heijnen [3]

[1] Department Special Needs Education, University of Oslo, 0318 Oslo, Norway
[2] Curtin School of Allied Health, Faculty of Health Sciences, Curtin University, Perth, WA 6102, Australia
[3] Department of Otorhinolaryngology and Head and Neck Surgery, Leiden University Medical Center, 1233 ZA Leiden, The Netherlands
[4] Department of Social Work, Education and Community Wellbeing, Faculty of Health & Life Sciences, Northumbria University, Newcastle upon the Tyne, NE7 7XA, United Kingdom
[5] Macquarie University, Department of Linguistics, Faculty of Medicine, Health, and Human Sciences, New South Wales 2109, Australia
[6] Boston University School of Medicine, Boston, MA 02118, United States
[7] Centre for Exercise and Active Rehabilitation, University of Greenwich SE9 2BP

Participants demographics

Participants demographics		Delphi Round I (N=78)		Delphi Round II (N=66)	
		n	%	*n*	%
**Continent of residence *(Number of countries)***	Europe (18 countries)	51	65.4	49	74.2
	Asia (7 countries)	7	9.0	5	7.6
	North America (2 countries)	4	5.1	2	3.0
	South America (2 countries)	7	9.0	3	4.5
	Oceania (2 countries)	9	11.5	7	10.6
**Highest qualification *(related to work in the field of dysphagia)***	Bachelor	12	16.2	9	13.6
	Master	19	25.7	24	36.4
	PhD	43	58.1	33	50.0
**Profession**	Speech Language Pathologist	46	59.0	41	62.1
	Occupational Therapist	6	7.7	5	7.6
	Dietician	1	1.3	1	1.5
	Medical Specialist	19	24.4	17	25.8
	Dual Allied Health / Med Specialist	6	7.7	2	3.0
**Practice setting**	Hospital / Teaching hospital	62	79.5	47	71.2
	University / Education sector	26	33.3	22	33.3
	Private practice	12	15.4	10	15.1
	Community health centre	7	9.0	8	12.1
	Residential aged / Disability care	6	7.7	2	3
	Student	5	6.5	5	7.6
**Patient populations**	Degenerative neurological disorders	65	83.3	52	78.8
	Non-degenerative / Acquired neurological trauma	64	82.1	54	81.1
	Geriatrics	34	43.6	28	42.4
	Oncology	32	41.0	34	51.5
	Respiratory diseases	23	29.5	18	27.3
	Gasteroenterology	17	21.8	14	21.2
**Years of experience**	5 - 10 yrs	17	21.8	15	22.7
	11 - 15 yrs	21	26.9	20	30.3
	16 - 20 yrs	14	17.9	11	16.7
	21 - 30 yrs	19	24.4	14	21.2
	> 30 yrs	7	9.0	6	9.1

Supplementary file S2.

Development of two patient self-reported measures on functional health status (FOD) and health-related quality of life (QOD) in adults with oropharyngeal dysphagia using the Delphi technique

Renée Speyer [1,2,3], Reinie Cordier [4,2], Deborah Denman [5], Catriona Windsor [1], Gintas P. Krisciunas [6], David Smithard [7] and Bas J. Heijnen [3]

[1] Department Special Needs Education, University of Oslo, 0318 Oslo, Norway
[2] Curtin School of Allied Health, Faculty of Health Sciences, Curtin University, Perth, WA 6102, Australia
[3] Department of Otorhinolaryngology and Head and Neck Surgery, Leiden University Medical Center, 1233 ZA Leiden, The Netherlands
[4] Department of Social Work, Education and Community Wellbeing, Faculty of Health & Life Sciences, Northumbria University, Newcastle upon the Tyne, NE7 7XA, United Kingdom
[5] Macquarie University, Department of Linguistics, Faculty of Medicine, Health, and Human Sciences, New South Wales 2109, Australia
[6] Boston University School of Medicine, Boston, MA 02118, United States
[7] Centre for Exercise and Active Rehabilitation, University of Greenwich SE9 2BP

Prototype measure 'Functional health status measure of Oropharyngeal Dysphagia' (FOD): *Example items*

I have difficulty swallowing thin liquids (e.g.: water, tea, coffee, juice, beer).

I avoid certain foods.

I take supplemental nourishment through a feeding tube.

I cannot maintain my weight.

It takes me a long time to eat.

I have difficulties chewing (these difficulties are not due to poor dentition).

There is food left in my mouth after I have swallowed.

Swallowing is more difficult when I am feeling tired.

Prototype measure 'health-related Quality of life measure of Oropharyngeal Dysphagia' (QOD): *Example items*

My swallowing difficulties make my life less enjoyable.

I feel anxious that I will choke when I eat.

I am embarrassed to eat or drink in the presence of other people.

Other people are irritated by my swallowing difficulties.

I feel excluded because of my swallowing difficulties.

My swallowing difficulties frustrate me.

Swallowing is stressful.

I feel emotionally worn out due to my swallowing difficulties.
